# Microfluidic Chip Fabrication of Fused Silica Using Microgrinding

**DOI:** 10.3390/mi14010096

**Published:** 2022-12-30

**Authors:** Pyeong An Lee, Ui Seok Lee, Dae Bo Sim, Bo Hyun Kim

**Affiliations:** 1 Department of Mechanical Engineering, Graduate School, Soongsil University, 369 Sangdo-ro, Dongjak-gu, Seoul 06978, Republic of Korea; 2 School of Mechanical Engineering, Soongsil University, 369 Sangdo-ro, Dongjak-gu, Seoul 06978, Republic of Korea

**Keywords:** microfluidic chip, fused silica, grinding

## Abstract

Although glass is in high demand as a material for microfluidic chips, it is still difficult to fabricate microstructures on glass. In this paper, polycrystalline diamond tools were fabricated through electrical discharge machining, and the microgrinding process for fused silica using the tools was studied. In order to improve the productivity, the machining effects of the high feed rate and depth of cut on the surface roughness of the channel bottoms and edge chipping were studied. A toolpath for the microchannels of a microfluidic chip was also studied and a microfluidic chip array was fabricated using this method.

## 1. Introduction

As a substrate material for microfluidic chips, many researchers have used polymer materials such as PDMS (polydimethylsiloxane) and PMMA (polymethyl methacrylate) because the manufacturing process is easy and the cost is low [1]. However, polymer-based microfluidic devices have some limitations in their applications due to their low mechanical, thermal and chemical stability [2,3,4].

Glass materials such as borosilicate, fused silica and sapphire can be alternative materials for microfluidic devices because they have many advantages such as excellent chemical resistance, high thermal stability, high mechanical strength and optical transparency [4,5,6]. However, they can be difficult to machine because of their mechanical properties such as their hardness and brittleness.

In order to process glass without cracks, in consideration of its optical properties, previous studies have focused on obtaining smooth surfaces via ductile-mode cutting. As a tool’s feed rate increases, the cutting force increases and cracks are more likely to occur. Therefore, studies involving the application of a low depth of cut or low feed rate have been conducted.

Takeuchi et al. experimentally verified that glass can be processed in a ductile mode using cutting processing [7]. Using a diamond ball-end mill, a three-dimensional shape with a diameter of 1 mm and a height of 30 µm was machined on BK7 glass, and a smooth surface with Ra 0.05 µm was obtained at a depth of cut of 0.1 µm. Matsumura and Ono used a cemented carbide ball-end mill to machine microchannels with a depth range of 15–20 µm in crown glass [8]. For ductile-mode cutting, the tool was tilted. The channel was machined without cracks by applying a feed rate of 8 µm/s and a depth of cut of 15 µm.

Polycrystalline diamond (PCD) is an electrically conductive material that can be easily machined via electrical discharge machining (EDM) [9,10,11]. Many researchers have used PCD microtools to machine brittle materials. Morgan et al. studied the machining of ultra-low expansion (ULE) glass using PCD tools with a diameter of 50 µm [12]. The tools were fabricated using EDM and a groove with a surface roughness of Ra 0.3 nm was machined under the machining conditions of a feed rate of 1 µm/s and a depth of cut of 0.1 µm. Perveen et al. machined microchannels in glass using a PCD tool [13]. In order to obtain a surface roughness of Ra 0.013 µm, a 2 µm depth of cut and 1 µm/s feed rate were applied. Katahira et al. machined microchannels in sapphire using a PCD tool with a diameter of 300 µm [14]. It was shown that a smooth surface of Ra 2.5 nm was machined under the machining conditions of a 0.2 µm depth of cut and 83 µm/s feed rate. Cao et al. [15] used electrochemical discharge machining (ECDM) and microgrinding to machine channels with a depth of 50 µm and a width of 60 µm in soda-lime glass.

Many researchers have studied the micromachining of glass. In the case of microfluidic chips, a large number of microchannels and microstructures are required, but there is a limit to increasing the machining speed while maintaining a high surface quality. In this study, microchannels on fused silica were machined for microfluidic chip fabrication and the machining characteristics were studied to increase the machining speed rather than to obtain a smooth surface.

## 2. Materials and Methods

**Figure 1:**
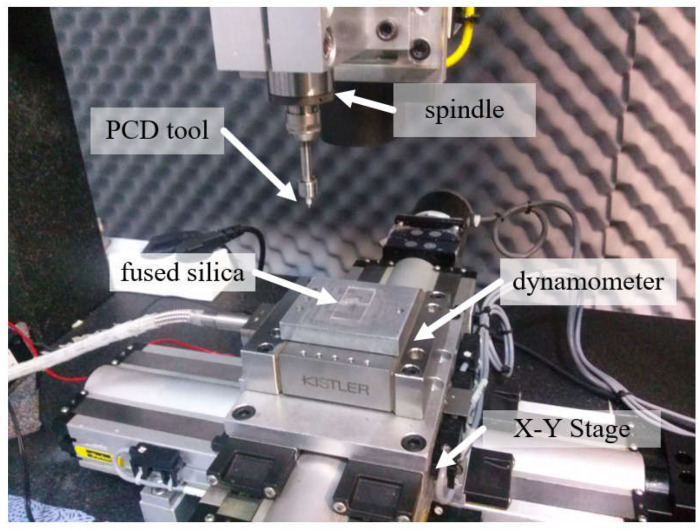
The microgrinding system.

**Figure 2:**
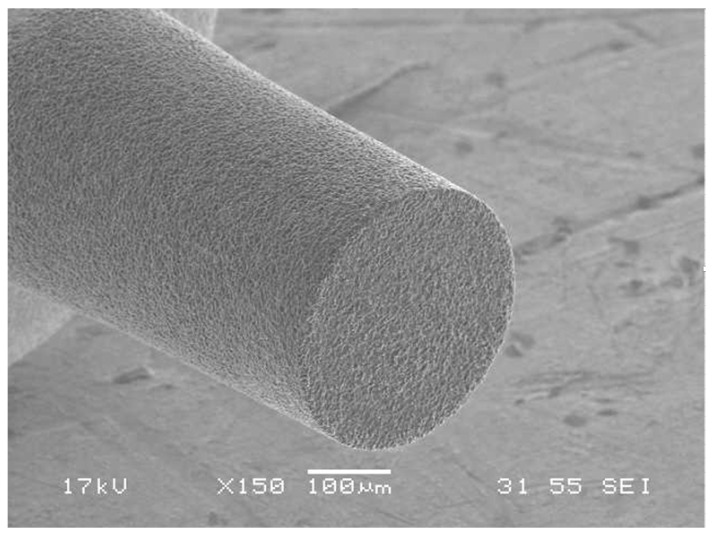
An SEM image of the microtool fabricated via WEDG (100 V, 5600 pF).

Figure 1 shows the experimental setup for the microgrinding process. The system consisted of an X–Y–Z stage, a high-speed spindle, and a dynamometer (9256C2, Kistler Instrumente AG, Winterthur, Switzerland) for measuring the cutting forces [10,11]. As a workpiece material, fused silica was used. The workpiece was a 15 mm × 15 mm plate with a thickness of 1 mm that was fixed on the dynamometer. Before the experiment, the spindle was operated for several tens of minutes to ensure a steady-state temperature distribution in the spindle system. Table 1 shows the properties of the fused silica. The system also had a wire electrodischarge grinding (WEDG) module for fabricating microtools. WEDG is one of the electrical discharge machining (EDM) methods using wire and can be used to fabricate microtools of various shapes [16]. An RC-type circuit consisting of resistors and capacitors was used as an EDM circuit. As the tool material, polycrystalline diamond (PCD) was used. Since PCD has a high hardness, it is suitable as a tool material for machining brittle materials such as glass, ceramics, cemented carbide and silicon carbide [17]. In this experiment, a microgrinding tool with a diameter of 300 µm fabricated via WEDG was used. Figure 2 shows an example of the PCD microtools. The surface roughness of the workpieces was measured using a laser confocal microscope (OLS5000, Olympus Corp., Tokyo, Japan). The average roughness (Ra) was used as a surface roughness parameter [18].

## 3. Results

A microchannel is one of the most important elements in a microfluidic chip. In order to minimize edge chipping and cracks that decrease the quality of the channel and to improve the machining speed, the machining characteristics under various conditions were analyzed. Microchannels were machined in fused silica by microgrinding and changes in the cutting force, surface roughness and edge chipping according to the feed rate, depth of cut and rotational tool speed were analyzed. The tool was fed in the vertical direction (z axis) by the depth of cut and then fed in the horizontal direction (x axis) to machine the channels layer-by-layer. The cutting force consisting of the normal force (Fz) and thrust force (Fx) was measured using a dynamometer. Table 2 shows the machining parameters.

### 3.1. Effect of Feed Rate

**Figure 3:**
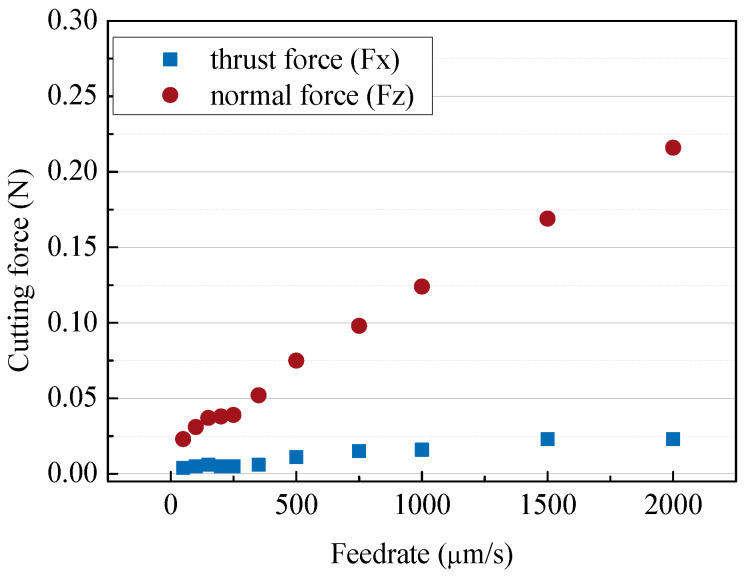
The cutting forces according to the different feed rates (feed rates: 10–2000 µm/s; depth of cut: 5 µm; 50,000 rpm).

**Figure 4:**
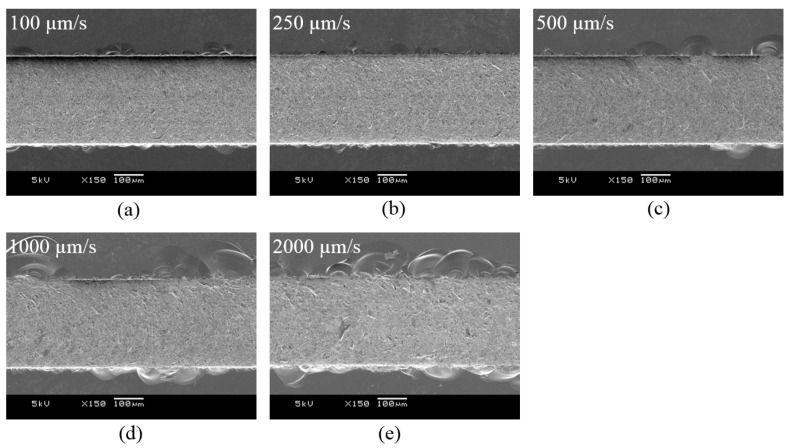
SEM images of the channels with different feed rates (**a**) 100 µm/s, (**b**) 250 µm/s, (**c**) 500 µm/s, (**d**) 1000 µm/s, (**e**) 2000 µm/s (depth of cut: 5 µm; 50,000 rpm).

**Figure 5:**
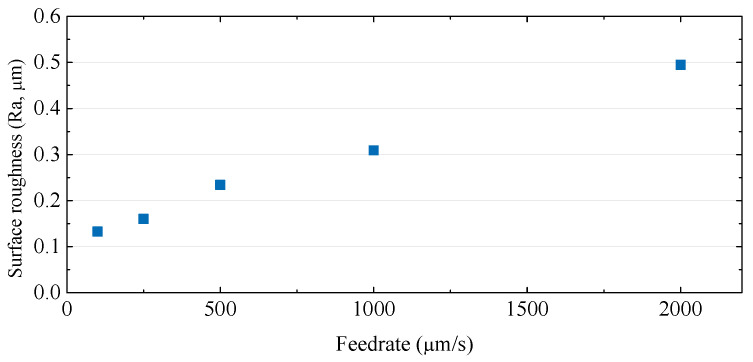
The surface roughness values of the channel bottoms according to the different feed rates (feed rates: 10–2000 µm/s; depth of cut: 5 µm; 50,000 rpm).

In order to improve the machining speed, a high feed rate is required, but as the feed rate increases, the cutting force increases and more cracks may occur. Cracks or edge chipping that occur on the machined surface degrade the quality of the microfluidic channels, so it is necessary to increase the feed rate while minimizing cracks. The experiment was conducted under the conditions of a depth of cut of 5 µm and a tool rotational speed of 50,000 rpm, and the feed rate range was from 10 µm/s to 2000 µm/s. Figure 3 and Figure 4 show the cutting force and SEM images of channels according to the tool feed rate. As the feed rate increased, the normal force (Fz) increased from 25 mN to 0.22 N. In Figure 3, it can be seen that the cutting directional force (Fx) was very low compared to the normal force, because the cutting depth was only 5 µm, which was as small as the tool edge radius. The tool was machined with a voltage of 100 V and a capacitance of 5600 pF and the tool had an edge radius of about 3–5 µm. As shown in Figure 4, edge chipping and bottom surface cracking also increased. As a result, as shown in Figure 5, the surface roughness gradually increased.

### 3.2. Effect of Depth of Cut

Since a depth of cut is a major factor affecting the material removal rate, the effect of the depth of cut was analyzed. The feed rate was set to 250 µm/s and the tool’s rotational speed was set to 50,000 rpm, while the depth of cut increased from 5 µm to 200 µm.

**Figure 6:**
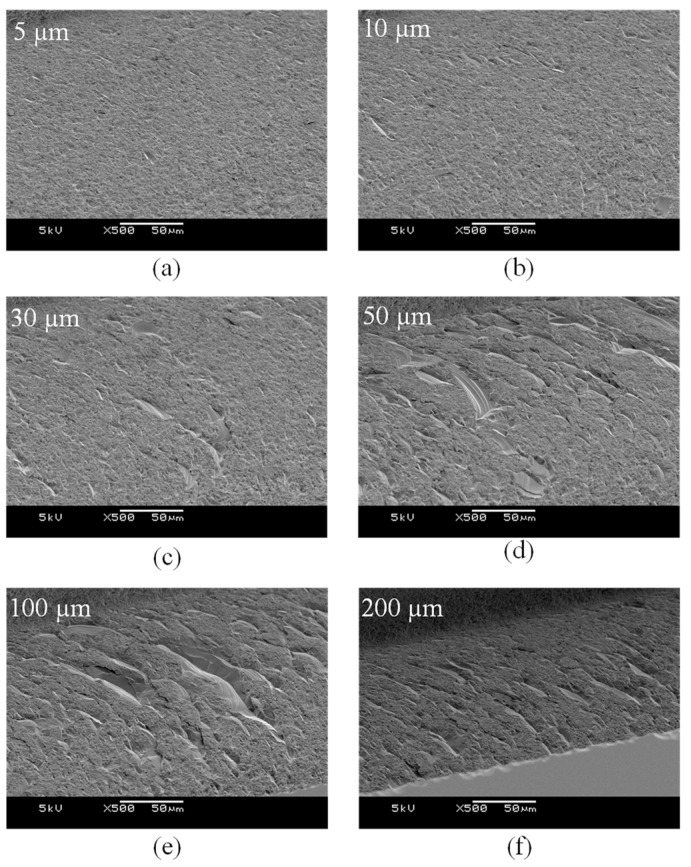
SEM images of the channels with different depths of cut (**a**) 5 µm, (**b**) 10 µm, (**c**) 30 µm, (**d**) 50 µm, (**e**) 100 µm, (**f**) 200 µm (feed rate 250 µm/s, 50,000 rpm).

**Figure 7:**
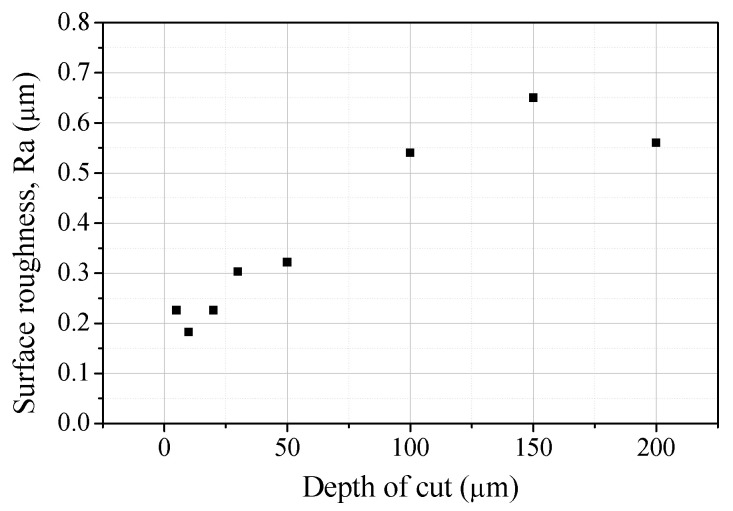
The surface roughness values of the channel bottoms according to the depth of cut.

Figure 6 and Figure 7 show SEM images and surface roughness values of the channel bottoms machined with different depths of cut. As the depth of cut increased, the size of the cracks on the bottom and the roughness increased. As the depth of cut and feed rate increased, the critical chip thickness increased [19]. As a result, more brittle fractures of the brittle materials occurred. A deep channel can be machined at once by applying a high depth of cut, but it is recommended to apply a depth of cut of 10 µm or less to suppress large cracks on the bottom of the channel.

**Figure 8:**
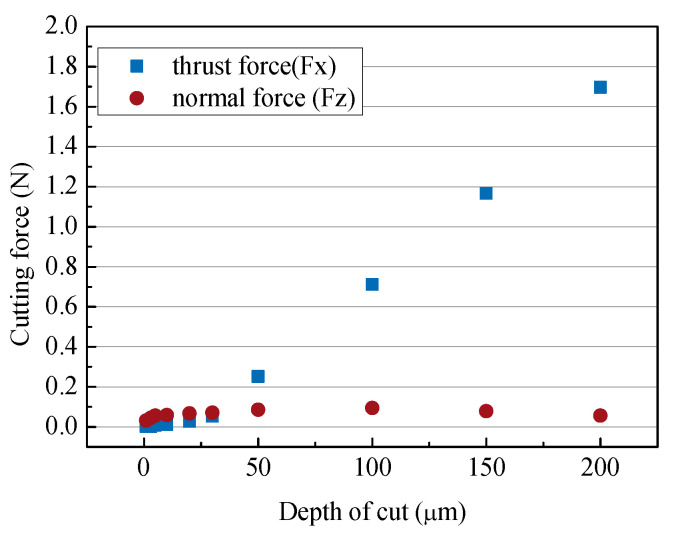
Cutting forces according to different depths of cut (depths of cut: 5–200 µm; feed rate: 250 µm/s; 50,000 rpm).

Figure 8 shows the change in cutting force according to the depth of cut. When the cutting depth was less than 30 µm, both the normal force and the cutting force were less than 0.1 N, but when the depth was greater than 50 µm, the cutting force became greater than the normal force. This was the effect of the size of the edge radius described above.

### 3.3. Effect of Rotational Speed

**Figure 9:**
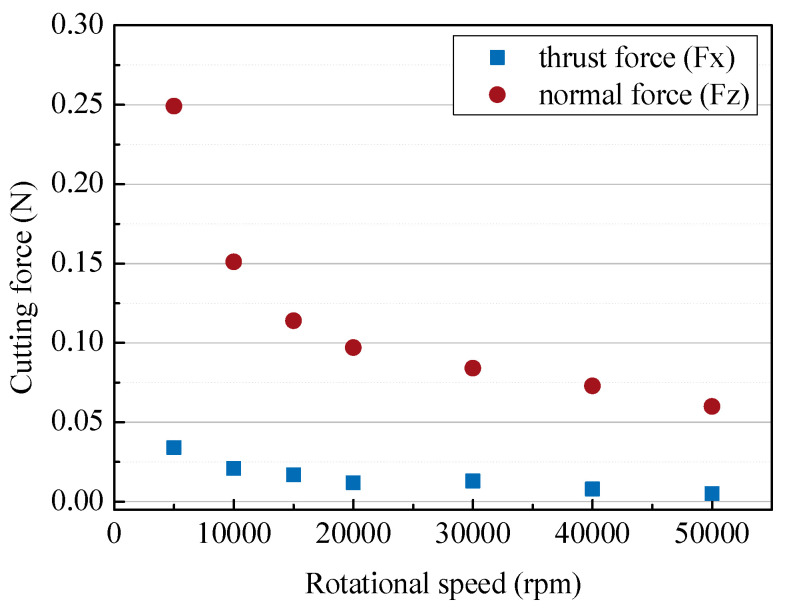
The cutting forces with different rotational tool speeds (250 µm/s; depth of cut: 5 µm).

In general, microtools require a high rotation speed because they have a small diameter and a low cutting speed. Figure 9 shows the changes in cutting force according to the rotational speed of the tool. As expected, the higher the rotational speed, the lower the cutting force. A high rotational speed of more than 30,000 rpm is required for a small cutting force of 0.1N, but this is also affected by other cutting conditions.

### 3.4. Tool Wear

**Figure 10:**
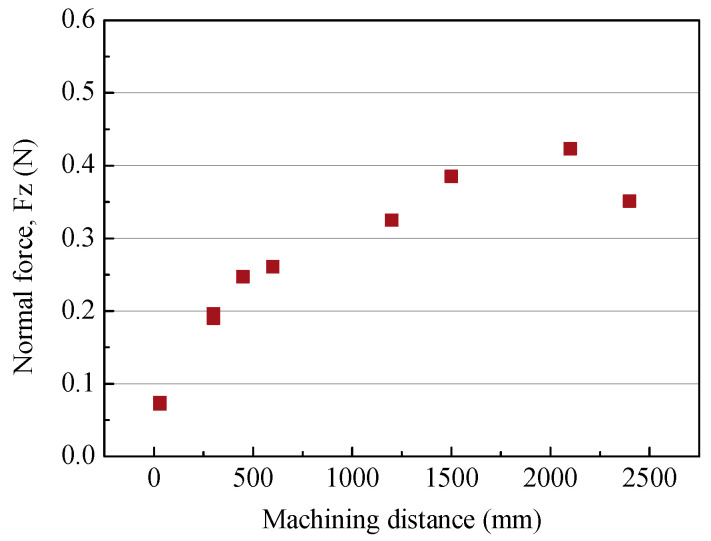
The cutting force according to the machining length.

**Figure 11:**
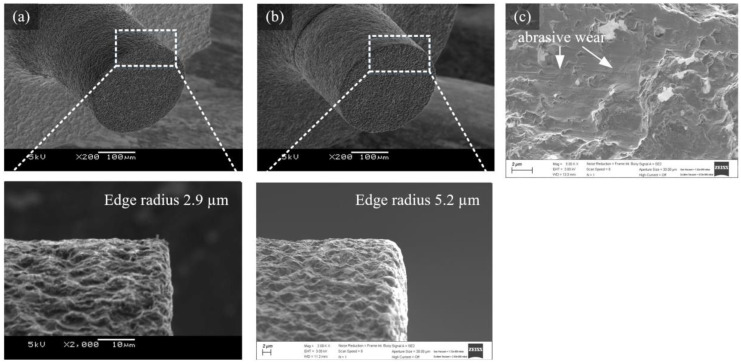
SEM images of a tool edge (**a**) before and (**b**) after machining and (**c**) abrasive wear on the tool’s bottom surface.

When tool wear occurs, the cutting force increases and the microtool can break. To analyze the tool wear characteristics, the cutting force and tool surface changes according to the machining distance were compared. A channel with a length of 2400 mm was machined under the conditions of a tool feed rate of 250 µm/s, a cutting depth of 5 µm and a tool rotational speed of 50,000 rpm. As the cutting length increased, the normal force continuously increased, as shown in Figure 10. Figure 11a,b show the tool before and after machining. It can be seen that the edge radius increased from 2.9 µm to 5.2 µm due to wear on the tool edge. Wear also occurred on the bottom surface near the edge of the tool. Figure 11c shows wear on the bottom surface of the tool edge. The rough surface generated via EDM was flattened by abrasive wear.

### 3.5. Microchannel

**Figure 12:**
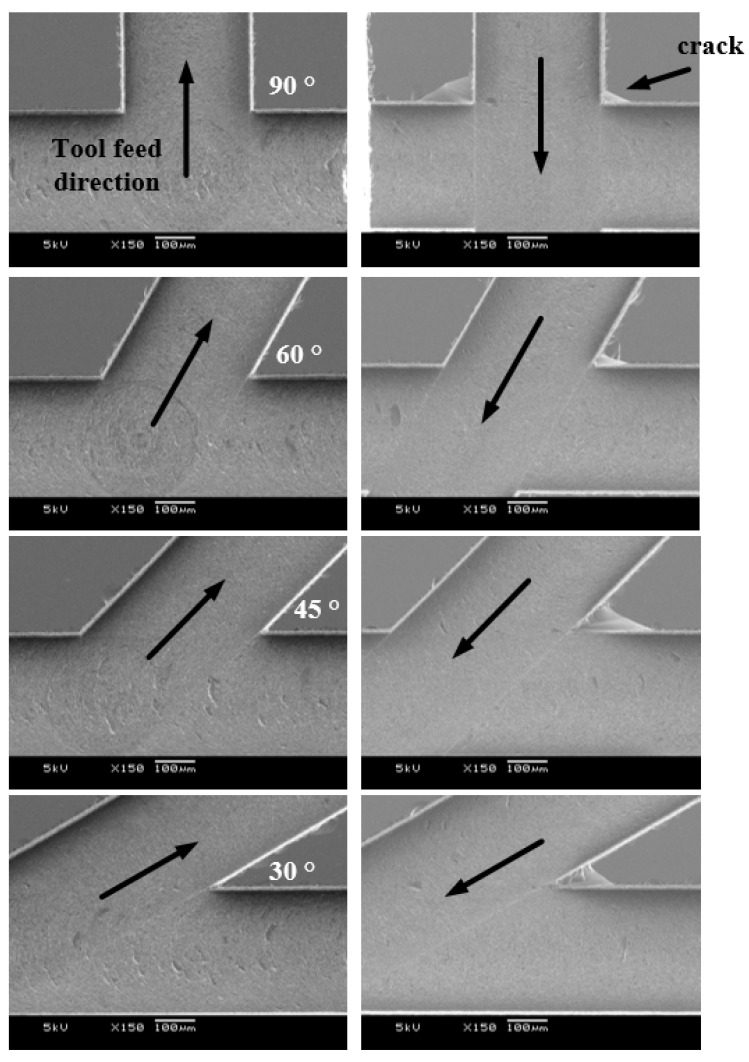
The SEM images of branched channels with different angles and tool feed directions (feed rate: 100 µm/s; depth of cut: 5 µm; 50,000 rpm; channel depth: 100 µm).

In general, microfluidic chips are composed of several branch channels used for injecting two or more fluids, and the angles between the channels are also varied. Micromixers, one of the applications of microfluidic chips, have several branch channels, and the lengths and angles between channels are very important for the efficiency of the fluid mixing [20,21,22]. When machining branch channels using microtools, the sizes of the cracks vary depending on the tool feed direction. To confirm this, branch channels with 30, 45, 60 and 90° angles were machined in different tool feed directions and cracks were observed, as shown in Figure 12. In the figure, the horizontal channel was machined first, then the branch channel was machined. As the tool moved from the first machined horizontal channel to the branch channel, the crack was suppressed at any angle. This was because compressive stress rather than tensile stress acted on the material in the channel. Therefore, it is necessary to generate tool paths considering the occurrence of cracks when machining branch channels.

**Figure 13:**
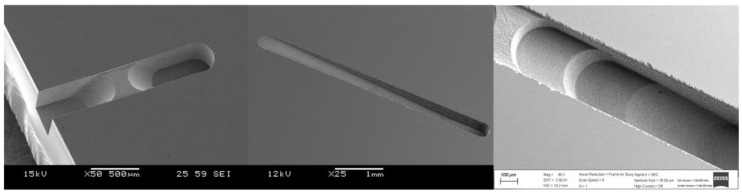
SEM images of channels with different slopes and depths.

**Figure 14:**
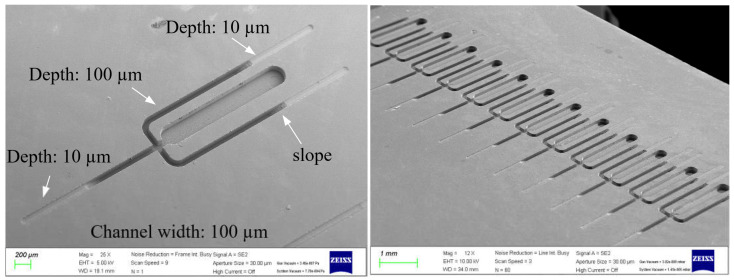
SEM images of microfluidic chip arrays with different channel depths (tool diameter: 100 µm; feed rate: 150 µm/s; channel depths: 10 and 100 µm).

One of the advantages of machining with microtools is that features with varying depths can be easily machined, similar to conventional CNC milling. Microchannels with various depths are difficult to make with conventional microfluidic chip processing techniques such as etching, powder blasting and laser machining. Figure 13 shows some examples of microchannels with varying slopes and depths. Figure 14 shows microchannel arrays with two depths, 10 µm and 100 µm. They were machined with a tool with a diameter of 100 µm and a feed rate of 150 µm/s. The machining time for one channel was about 30 min to 1 h, depending on the machining conditions.

## 4. Discussion and Conclusions

The microgrinding of fused silica for the machining of the microchannels of a microfluidic chip was studied. In order to increase productivity, the machining characteristics were analyzed by applying a high feed rate and depth of cut. At a feed rate of 250 µm/s, a tool rotation speed of 50,000 rpm and a depth of cut of 5 ~ 10 µm, the microchannel could be machined without large edge chipping. Cracks occurred on the bottom surface under all conditions, but the surface roughness of the bottom surface was up to 0.2 µm Ra.

Microgrinding with a PCD tool can be used to machine microchannels without a complicated process as compared to conventional methods. Since various tool shapes can be made via WEDG, not only square cross-sections but also triangular and semi-circular cross-sections of the channels can be obtained, and channels of various depths can be machined. In addition, not only channels but also microholes can be machined with a single tool.

**Figure 15:**
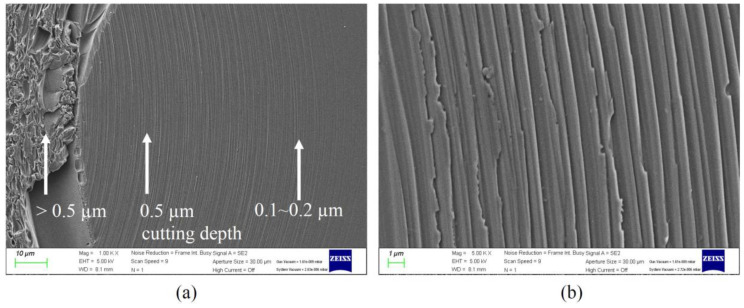
(**a**) The channel bottom machined with varying cutting depths and (**b**) an enlarged view of the channel bottom.

Although cracks occur at the channel bottom, the surface quality can be improved and the roughness can be reduced to 0.067 µm Ra using post-processing methods such as laser polishing [23]. When the depth of cut is extremely low, a surface without cracks and with excellent surface quality can be obtained. However, since the depth of cut should be lower than 1 µm, more research on this is needed to improve the productivity. Figure 15a shows the machined surfaces according to the cutting depth, and at a cutting depth of 0.5 µm or less, as shown in Figure 15b, the surface was machined in ductile mode. The average roughness was 0.017 µm Ra.

The tool wear is also a very important machining characteristic. In this study, it was possible to machine pieces at up to several thousand mm without severe tool wear. Although the tool wear will increase with larger amounts of machining, if only the bottom of the tool is re-machined via EDM (tool dressing), the tool can continue to be used. For this, it is important to use a simple tool such as in this study, where it took less than 1 min to dress the tool. Compared to the conventional methods, microgrinding using microtools involves a low machining speed and is not suitable for mass production. However, due to the simple process and low cost, it seems to be useful for prototyping to determine the channel design.

## Figures and Tables

**Table 1:** The physical properties of the fused silica.

Properties	Value
density (g/cm^3^)	2.2
Young’s modulus (GPa)	71.6
Poisson’s ratio	0.17
torsional rigidity	31.4
compression strength (GPa)	1.1
bending strength (MPa)	69
tensile strength (MPa)	55
Vickers hardness	8.8–10.1

**Table 2:** The grinding parameters for the fused silica.

Parameters	Value
tool diameter	300 µm
tool rotational speed	2000–50,000 rpm
feed rate	10–2000 µm/s
depth of cut	5–200 µm
